# Selfish Little Circles: Transmission Bias and Evolution of Large Deletion-Bearing Mitochondrial DNA in *Caenorhabditis briggsae* Nematodes

**DOI:** 10.1371/journal.pone.0041433

**Published:** 2012-07-31

**Authors:** Katie A. Clark, Dana K. Howe, Kristin Gafner, Danika Kusuma, Sita Ping, Suzanne Estes, Dee R. Denver

**Affiliations:** 1 Department of Zoology and Center for Genome Research and Biocomputing, Oregon State University, Corvallis, Oregon, United States of America; 2 Department of Biology, Portland State University, Portland, Oregon, United States of America; Centre National de la Recherche Scientique & University of Nice Sophia-Antipolis, France

## Abstract

Selfish DNA poses a significant challenge to genome stability and organismal fitness in diverse eukaryotic lineages. Although selfish mitochondrial DNA (mtDNA) has known associations with cytoplasmic male sterility in numerous gynodioecious plant species and is manifested as petite mutants in experimental yeast lab populations, examples of selfish mtDNA in animals are less common. We analyzed the inheritance and evolution of mitochondrial DNA bearing large heteroplasmic deletions including *nad5* gene sequences (*nad5*Δ mtDNA), in the nematode *Caenorhabditis briggsae*. The deletion is widespread in *C. briggsae* natural populations and is associated with deleterious organismal effects. We studied the inheritance patterns of *nad5*Δ mtDNA using eight sets of *C. briggsae* mutation-accumulation (MA) lines, each initiated from a different natural strain progenitor and bottlenecked as single hermaphrodites across generations. We observed a consistent and strong drive toward higher levels of deletion-bearing molecules in the heteroplasmic pool of mtDNA after ten generations of bottlenecking. Our results demonstrate a uniform transmission bias whereby *nad5*Δ mtDNA accumulates to higher levels relative to intact mtDNA in multiple genetically diverse natural strains of *C. briggsae*. We calculated an average 1% per-generation transmission bias for deletion-bearing mtDNA relative to intact genomes. Our study, coupled with known deleterious phenotypes associated with high deletion levels, shows that *nad5*Δ mtDNA are selfish genetic elements that have evolved in natural populations of *C. briggsae*, offering a powerful new system to study selfish mtDNA dynamics in metazoans.

## Introduction

Deleterious mutations leading to reduced organismal fitness are expected to be eliminated from natural populations due to purifying selection, provided selection is sufficiently strong. However, some genetic elements, termed “selfish” or “parasitic” DNA, display transmission advantages and long-term evolutionary persistence despite neutral or negative organismal fitness consequences. Examples include homing endonucleases, supernumerary chromosomes, segregation distorters, transposable elements, and the cytoplasmic genomes of microorganisms and organelles [1]. Although mitochondrial DNA (mtDNA)-encoded and nuclear electron transport chains genes have coevolved in eukaryotes to produce functional electron transport chain complexes, mtDNA also underlies cytonuclear conflict and cytoplasmic male sterility in over 150 plant species [2]. *Saccharomyces cerevisiae* petite mutants, caused by heteroplasmic mtDNA molecules bearing large deletions, were found to predominate in small but not large experimental yeast populations [3]. Selfish mtDNA dynamics have also been characterized in species of filamentous fungi [4], [5]. These findings indicate that mtDNA is capable of selfish evolutionary dynamics in certain contexts.

mtDNA bearing large function-disrupting deletions are well-known to accumulate in the aging somatic tissues of many animal species [6], [7], [8], but rarely transmit to progeny through the germline so are not generally considered from selfish DNA perspectives. There is only one reported case of a transmitted large mtDNA deletion in humans [9]. One exception to this generalization is the nematode *Caenorhabditis briggsae*. In this species, many natural lineages are known to encode large heteroplasmic mtDNA deletions that eliminates 786 bp, including many highly conserved codons, from the 5′ end of the *nad5* gene [10]. This mtDNA deletion (*nad5*Δ) is associated with 21-bp direct repeats, one motif in the *nad5* gene and the second present in an upstream pseudogene element (named Ψnad5-2) which is homologous to and presumably derived from *nad5* through illegitimate recombination mechanisms [11]. Different natural strains of *C. briggsae* vary in the relative abundances of deletion-bearing versus intact mitochondrial genomes, ranging from zero to ∼60% deletion-bearing molecules [10]. Negative relationships between heteroplasmic *nad5*Δ mtDNA levels and strain-specific fecundity and pharyngeal pumping rates [12] indicate that these deletions are deleterious to organismal fitness under lab conditions. Mitochondria from high-deletion *C. briggsae* strains produce significantly more damaging reactive oxygen species (ROS) than low-deletion strains [12]. These findings suggest that *nad5*Δ mtDNA molecules have negative organismal effects, likely as a consequence of dysfunction in electron transport chain Complex I, of which *nad5* encodes an integral subunit.

Phylogenetic analyses of *C. briggsae* natural strain mtDNA sequences reveal the presence of three major intraspecific clades that, to some extent, exist in distinct latitudinal ranges [11]. Analyses of *C. briggsae* nuclear loci show similar latitudinal phylogeographic patterns [13], [14]. *C. briggsae* Clade I (a.k.a. the “tropical” clade) is composed of genetically diverse strains found in tropical latitudes including strain AF16 which serves as the common lab strain with a reference nuclear genome sequence available [15]. Clade II (a.k.a. the “temperate” clade) is composed of strains found in northern latitudes; there is very little genetic diversity in Clade II, which has led to the hypothesis that these strains invaded temperate latitudes in the very recent evolutionary past [13]. Clade III (a.k.a. the “equatorial” clade) was originally identified based on a small number of strains from Kenya with highly divergent mtDNA and nuclear sequences relative to Clade I and II strains, though more members of this clade have recently been discovered in India [11].

**Figure 1:**
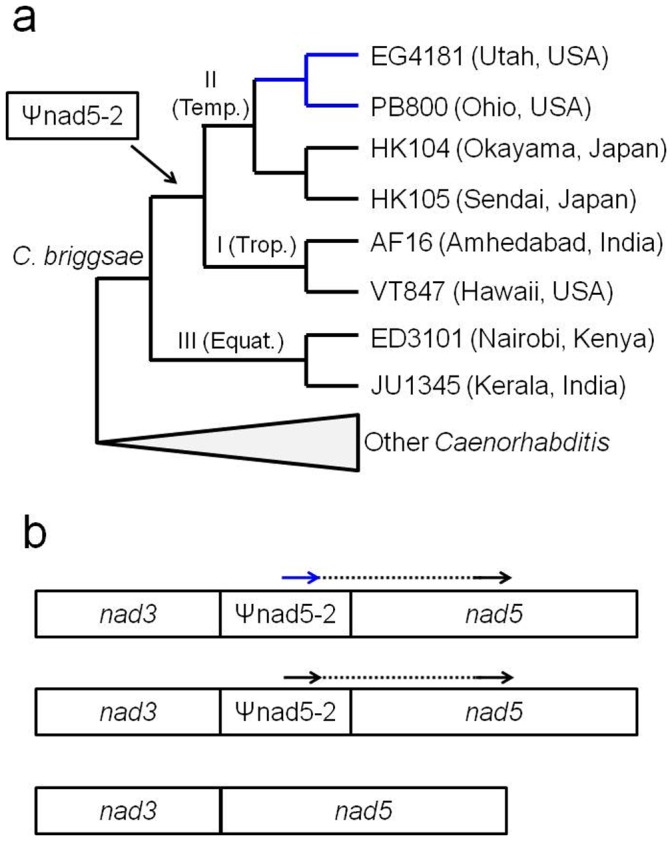
Phylogenetic relationships based on mtDNA of the eight *C. briggsae* natural strains used for MA line progenitors (a) and schematic of the *nad5*Δ locus (b). In (a), the three major intraspecific mtDNA clades and origin of the Ψnad5-2 element are indicated next to the relevant internal branches. The blue branches lead to MA line progenitors that encode the putative compensatory mutations associated with the *nad5*Δ locus [10]. In (b), the arrows indicate positions of the 21-bp direct repeats associated with deletion formation. The dashed line indicates DNA sequences missing in canonical *nad5*Δ molecules. The blue arrow indicates the position of the direct repeat bearing putative compensatory mutations. Clade III strains lack Ψnad5-2 elements and associated deletions, as indicated in the bottom mtDNA gene model.

The three *C. briggsae* intraspecific clades vary in terms of genotypes at the *nad5*Δ locus surveyed here. The Ψnad5-2 element responsible for *nad5*Δ mtDNA is present in Clade I and II strains, but not Clade III strains [10]; thus, Clade III nematodes do not experience deletion events at this locus. All Clade I and some (e.g., HK104, HK105) Clade II strains encode perfect 21-bp direct repeats at the deletion locus. Some Clade II strains (e.g., EG4181, PB800), however, encode imperfect 21-bp direct repeats due to the presence of polymorphisms in the Ψnad5-2 direct repeat unit (Figure 1). Natural strains encoding such imperfect repeats have been shown to contain lower heteroplasmic levels of *nad5*Δ mtDNA as compared to those with perfect repeats [10]. Thus, the polymorphisms resulting in imperfect repeat units in these Clade II nematodes are hypothesized to be compensatory in nature, resulting in lower levels of the presumably deleterious deletions.

Are *C. briggsae nad5*Δ mtDNA molecules selfish elements? Genetic elements are characterized as selfish only if they meet two key criteria [1]. First, the element must have a transmission advantage relative to other DNA encoded in the organism. Second, the elements must be neutral or deleterious to organismal fitness. Although there is ample evidence that the deletions are deleterious, the transmission dynamics of *nad5*Δ mtDNA in *C. briggsae* have not been characterized.

We investigated the inheritance patterns of *C. briggsae nad5*Δ mtDNA using a mutation-accumulation (MA) line approach. Eight different sets of MA lines were examined, initiated from distinct natural strain progenitors that varied in terms of heteroplasmic *nad5*Δ mtDNA levels. Our findings revealed a highly unstable and dynamic nature of *C. briggsae* deletion heteroplasmy with a uniform bias toward the inheritance of increasing levels of deletion-bearing mtDNA across ten generations.

## Results

### *C. briggsae* MA Lines: Rationale and Experimental Design

We applied a MA line approach [16] to investigate patterns of mtDNA inheritance in diverse natural *C. briggsae* genotypes. Eight *C. briggsae* natural strains were chosen to serve as progenitor strains to include representatives of the three major intraspecific *C. briggsae* clades, as well as strains that vary in terms of relevant genotypes at the *nad5* locus surveyed here (Figure 1). For each of the eight *C. briggsae* progenitors shown in Figure 1a, we initiated 24 distinct MA lines. Each of the eight sets of MA lines was initiated from the progeny of a single hermaphrodite individual. For each of the eight focal strains, we picked a single hermaphrodite nematode at the L4 larval stage which was allowed to self-reproduce. From these self-progeny, 24 L4-stage nematodes were individually picked to separate plates – these individuals constituted MA line progenitors (G0). After these G0 progenitors self-reproduced and the first set of progeny nematodes was transferred to new plates (leading to G1), the remainder of the F_1_ progeny on the G0 plate were allowed to undergo another round of self-reproduction (leading to F_2_ self-progeny) and were subsequently harvested for PCR analysis (representative of G0). The MA lines were bottlenecked as single L4-stage hermaphrodites in the lab for ten generations (G10). The individual nematodes selected to initiate G10 were allowed to self-reproduce for two generations (as with G0), after which nematodes were harvested for PCR analysis.

**Figure 2:**
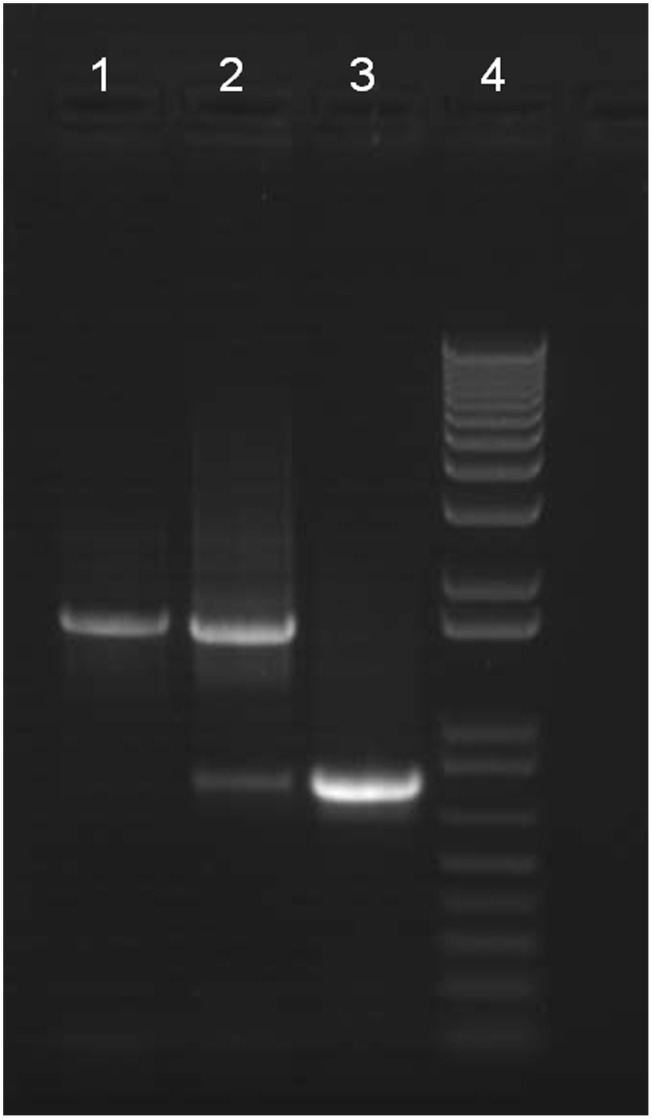
Gel electrophoresis analysis of *nad5*Δ mtDNA. *C. briggsae* DNA samples were analyzed by PCR using primers flanking the *nad5*Δ locus. Resultant PCR products were subject to agarose gel electrophoresis and scored according to the PCR band genotypes described in the Results. Lane 1 shows a sampled scored as intact (large band only); lane 2 shows a sample scored as intermediate (both large and small bands); lane 3 shows a sample scored as deletion (small band only). Lane 4 shows the molecular marker (1 kb+ DNA ladder).

We applied a standard PCR assay previously used [10] to assess the relative levels of intact and *nad5*Δ mtDNA in a biological sample. The assay involves two PCR primers (Cb_mt1F positioned in the *nad3* gene, 58R in the *nad5* gene) that flank the Ψnad5-2 pseudogene element (in strains where present) and its associated deletion products (Figure 2). In Clade III *C. briggsae* strains that lack Ψnad5-2 and associated *nad5*Δ mtDNA, this PCR produces a single amplicon product as visualized by agarose gel electrophoresis, as expected. In Clade I and II strains where the Ψnad5-2 element and associated *nad5*Δ mtDNA occur, the reaction has the potential to produce two resultant amplicons of different sizes. A larger amplicon can be produced that contains all of the *nad3*, Ψnad5-2, and *nad5* sequences present in an intact mtDNA molecule. A smaller amplicon, containing only the sequences that flank deletions in deletion-bearing molecules, results from deletion-bearing mtDNA. Thus, for this PCR assay, there were three categorical results possible: large band only (we call this the “intact” PCR genotype), both large and small band (“intermediate” genotype), and small band only (“deletion” genotype). In our first report of the *C. briggsae nad5*Δ locus [10], we performed a comparative analysis of *nad5* deletion level estimations based on a quantitative PCR (qPCR) approach in relation to results from the previously described standard PCR band scoring assay. This analysis revealed a significant positive correlation (Spearman rank correlation  = 0.74, *P*<10^−15^) between *nad5*Δ mtDNA frequencies as estimated by qPCR and the categorical results of the standard PCR assay. DNA samples that lead to an intact PCR band genotype result in standard PCR assays yielded an average qPCR deletion frequency estimate of 0.04 (±0.06) for that DNA sample (i.e., 4% of total mtDNA estimated to bear deletions). DNA samples resulting in intermediate PCR band genotypes yielded an average qPCR deletion frequency estimate of 0.30 (±0.15). DNA samples resulting in deletion PCR band genotypes yielded an average qPCR deletion frequency estimate of 0.60 (±0.07). Although the intermediate category is broad, corresponding to qPCR deletion frequency estimates ranging from ∼15 to 45%, earlier attempts to subdivide this broad category (e.g., ‘intact predominant’, ‘deletion predominant’) in our previous study [10] resulted in data that did not significantly correlate with corresponding qPCR estimates of deletion levels. Thus, we implemented the three-category band scoring approach. Given the consistency in results between the more sensitive qPCR assay and the coarser standard PCR assay, we concluded that the standard PCR approach constituted an effective, rapid and inexpensive method for estimating the general levels of deletion-bearing molecules in *C. briggsae* DNA samples, and applied the assay to the experiments reported here.

### Variation in *nad5*Δ mtDNA Levels Among MA Line Progenitors

**Figure 3:**
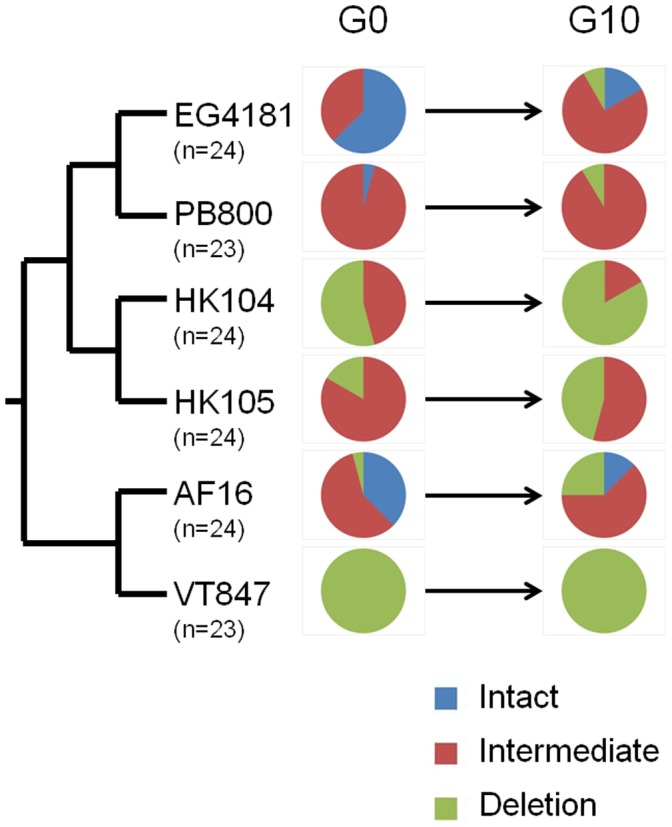
Changes in *nad5*Δ mtDNA levels across ten generations in MA lines. The pie charts illustrate changes in the relative proportions of the three *nad5*Δ locus PCR band genotypes among the MA lines analyzed from a given *C. briggsae* strain. G0 indicates the proportions observed in MA line-specific progenitor samples; G10 indicates proportions observed after ten bottleneck generations. Numbers of MA lines analyzed are indicated below strain names.

We first analyzed variation in *nad5*Δ mtDNA levels among the samples representing different MA line-specific progenitors (G0) using the aforementioned standard PCR band gel scoring approach. For all MA line progenitors derived from the two Clade III strains, a single band was identified of the expected size (slightly smaller than intact bands from Clade I and II strains). However, we observed substantial G0 variation both among the six MA line progenitor genotypes from Clade I and II, and among replicate lines of a common progenitor genotype at the *nad5* locus (Figure 3). Strain AF16 from Clade I showed all three PCR band genotypes (intact, intermediate, deletion) present among the 24 AF16 progenitor lines assayed. Four progenitor strains, all from Clade II, displayed two PCR band genotypes among the progenitor G0 samples assayed. Strain VT847 from Clade I showed the least amount of variation at GO with 23/23 progenitors assayed scored as deletion. These findings demonstrate that different *C. briggsae* nematodes of a common strain can vary substantially in terms of *nad5*Δ mtDNA levels.

### Changes in *nad5*Δ mtDNA Levels Across Generations

Along with standing variation in *nad5*Δ mtDNA levels observed within and between *C. briggsae* MA line progenitors at G0, we also observed variation in *nad5* deletion PCR genotypes between G0 and G10 in the MA lines (Table 1). In total, among the 142 MA lines analyzed at both G0 and G10, 37 (26%) experienced detectable *nad5*Δ mtDNA level shifts between the ten bottleneck generations. All of the changes observed were from a state of lower *nad5*Δ levels at G0 to higher deletion levels at G10. Among the 37 total G0 to G10 changes observed at the *nad5* locus, 14 were from intact to intermediate PCR band genotypes, 19 were from intermediate to deletion, and four were from intact to deletion. EG4181 showed the greatest degree of change between G0 and G10 with 46% of MA lines scored differently at G0 and G10. VT847 showed no change from G0 to G10, with all MA lines scored as deletion both at G0 and G10. Clade III progenitors (ED3101, JU1345) all displayed the single expected band at G0 and G10 associated with their lack of the Ψnad5-2 element and associated deletion products. The uniform drive toward higher deletion levels across ten bottleneck generations is also reflected in changes in the relative proportions of the three *nad5* PCR genotypes observed at G0 versus G10. Among the 142 *C. briggsae* Clade I and II samples assayed at G0, 18% were scored as intact, 53% were scored as intermediate and 29% were scored as deletion at this locus. At G10, however, 5% were scored as intact, 50% as intermediate, and 45% as deletion. These results demonstrate a clear and strong bias toward the accumulation of higher *nad5*Δ mtDNA levels across short generational intervals and under extreme bottlenecking.

**Table 1:** Changes in *nad5Δ* PCR band genotype from G0 to G10.

Strain	staticintc	static intm	staticdel	↑*nad5Δ* intc→intm	↑*nad5Δ* intc→del	↑*nad5Δ* intm→del	↓*nad5Δ* intm→intc	↓*nad5Δ* del→intm	↓*nad5Δ* del→intm	total
EG4181	4	9	0	9	2	0	0	0	0	24
PB800	0	20	0	1	0	2	0	0	0	23
HK104	0	4	13	0	0	7	0	0	0	24
HK105	0	13	4	0	0	7	0	0	0	24
AF16	3	11	1	4	2	3	0	0	0	24
VT847	0	0	23	0	0	0	0	0	0	23

Static indicates cases where *nad5Δ* mtDNA levels stay the same, ↑*nad5Δ* indicates cases where levels increase, and ↓*nad5Δ* indicates cases where levels decrease. intc indicates the intact PCR band category, intm indicates the intermediate band category, and del indicates the deletion band category.

**Figure 4:**
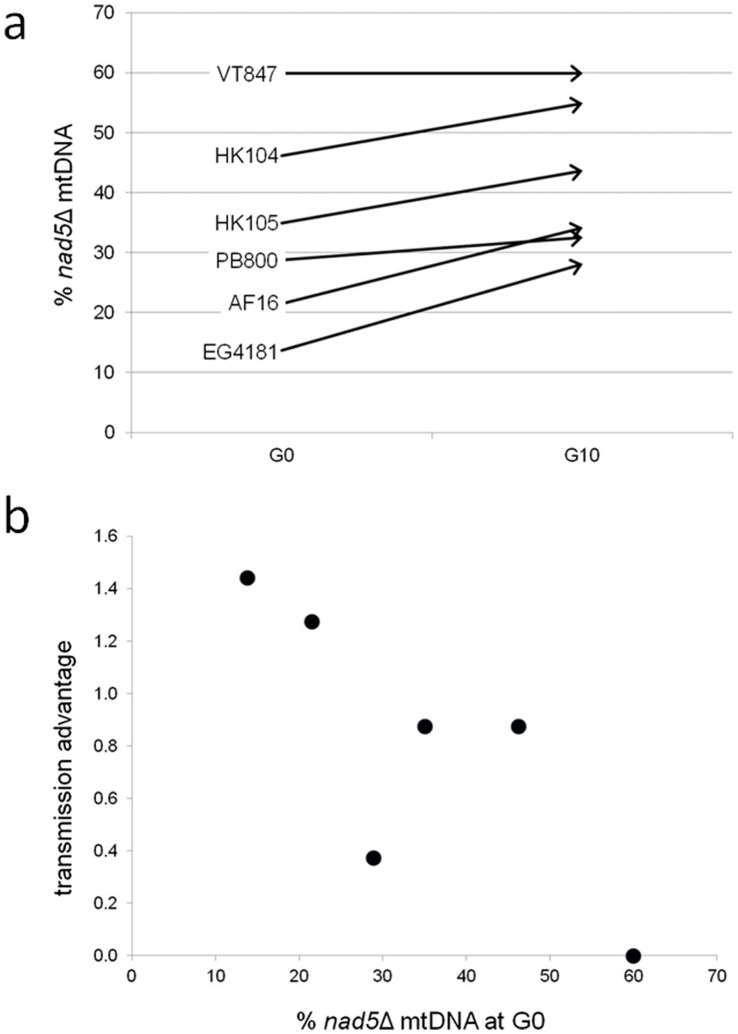
Transmission bias of *nad5*Δ mtDNA. (a) For each of the six Clade I and II C. briggsae strains analyzed, the change in average estimated percent *nad5*Δ mtDNA between G0 and G10 is shown. (b) The per-generation percent transmission advantage is shown as a function of estimated %*nad5*Δ mtDNA at G0.

We next quantified a rough estimate of the per-generation transmission advantage of the *nad5*Δ mtDNA for each of the six relevant (Clade I and Clade II) *C. briggsae* strains. This was accomplished by comparing the averaged estimates of *nad5*Δ mtDNA levels at G0 and G10 for each of the strains (Figure 4). For 5/6 strains, average *nad5*Δ levels increased across the ten generations, ranging from a ∼4% (PB800) to a ∼14% (EG4181) increase. The sole exception was strain VT847 which displayed deletion band PCR genotypes for all G0 and G10 samples analyzed. Thus, our findings suggest per-generation transmission biases ranging from 0.4 to 1.4% for *nad5*Δ mtDNA among five *C. briggsae* natural strains. The overall average per-generation transmission bias for all *C. briggsae* lines analyzed was 1.0%. We also compared strain-specific *nad5*Δ mtDNA transmission biases to starting *nad5*Δ mtDNA levels at G0 (Figure 4b) and discovered a negative correlation between these two variables (Spearman rank correlation = −0.80) which was weakly significant (*P*  = 0.05).

A two-way ANOVA statistical characterization of *nad5*Δ mtDNA levels at G0 and G10 in the six relevant *C. briggsae* strains revealed a highly significant effect of both strain (*P* = 2.2×10^−45^) and generation (*P* = 5.9×10^−9^) – see Table 2. These results indicate that *nad5*Δ mtDNA levels varied significantly both as a function of the *C. briggsae* strain and between G0 and G10. A significant (strain×generation) interaction effect was also revealed by our analysis (*P*  = 0.005), indicating significant variation among the strains in *nad5*Δ levels between G0 and G10.

**Table 2:** ANOVA results for deletion level variation among strains and generations.

Source of Variation	SS	df	MS	F	P-value	F crit
Sample (Strain)	5.03399	5	1.006798	67.80897	2.18E-45	2.248208
Columns (Generation)	0.537509	1	0.537509	36.20181	5.92E-09	3.876923
Interaction (Strain x Gen.)	0.253074	5	0.050615	3.408962	0.005301	2.248208
Within	3.919757	264	0.014848			
Total	9.744329	275				

### DNA Sequencing Characterization of Deletion Products

We carried out direct fluorescent capillary DNA sequencing analysis of the observed deletion products from a subset of the MA lines to determine whether the specific deletions observed were common among lines or if new deletions appeared during the experiment. We targeted select MA lines where deletion levels increased from G0 to G10, transitioning from intermediate to deletion PCR genotypes at the *nad5*Δ locus. Seventeen total MA lines were analyzed, including representatives from AF16 (n = 3), HK104 (n = 7), HK105 (n = 6), and PB800 (n = 1). In most cases, however, sequence data was obtained for the G10 samples only; the deletion bands present in the G0 samples for most lines analyzed provided insufficient template DNA after gel extraction (required to separate from intact bands) for successful sequencing reactions.

For AF16, one line was analyzed at both G0 and G10; the deletion product sequences in both samples were identical and the same as that originally reported for *C. briggsae* natural strains [10] (referred to herein as the ‘canonical’ deletion). The two other AF16 lines examined yielded successful sequencing reactions at G10 only, and each yielded a novel deletion sequence. One product contained an additional ∼108 bp of *nad5* gene sequence; the other product contained 5 bp of additional *nad5* sequence (both relative to canonical deletion sequence boundaries). Estimates for additional amounts of *nad5* sequences are approximate due to uncertainties associated with high sequence homology between the *nad5* gene and the Ψnad5-2 element (responsible for deletion formation). For HK104, two lines were analyzed at G0 and G10 and both samples harbored the canonical deletion boundaries at both generations. We also note here that for one of these lines a base substitution mutation was observed in this region at G10. Five additional HK104 MA lines yielded data for G10 only, revealing the canonical deletion in all cases. The six HK105 MA lines were analyzed at G10 only and 4/6 revealed the canonical deletion sequence boundary. One of these four sequences contained a base substitution polymorphism relative to the other three sequences. The other two HK105 G10 sequences revealed a novel deletion shared by the two lines, containing ∼107 bp of additional *nad5* DNA sequence relative to the canonical deletion. The sole PB800 sample evaluated was analyzed at G10 only and contained the canonical deletion sequence. Thus, in 13/17 MA lines analyzed the originally reported canonical deletion boundary was observed at G10 (and at G0 in those cases where data was available). Three other deletion types were observed, all containing additional *nad5* gene sequence relative to the canonical deletion.

## Discussion

The first criterion required to characterize a particular piece of DNA as a selfish genetic element is that it must have a transmission advantage over other DNA molecules in the organism. In this study, we observed that *nad5*Δ mtDNA molecules accumulate to higher relative levels over intact mtDNA after ten generations of bottlenecking in MA lines (Figure 3, Figure 4, Table 1). Among the 142 MA lines derived from Clade I and II progenitors analyzed, 37 were found to have increased *nad5*Δ mtDNA levels across ten generations and zero had decreased relative deletion levels that were detectable by our PCR assay. The observed accumulation of higher *nad5*Δ mtDNA levels in the MA lines might be a consequence of a replicative advantage for these molecules over intact mtDNA, as has been suggested and demonstrated in other systems [17], [18]. A second (and not mutually exclusive) possibility is that there is a high rate of *de novo* mtDNA deletion formation at this locus, presumably through illegitimate recombination mechanisms, that contributes to the higher observed *nad5*Δ levels at G10. Our DNA sequencing analysis revealed that the majority of lines analyzed at G10 (13/17) harbored the canonical *nad5Δ* deletion boundary originally reported for *C. briggsae* [10]. Three difference deletion boundaries were detected in our study (one boundary shared by two different MA lines). These cases might reflect *de novo* deletion events that occurred and then drifted to high levels during the ten bottleneck generations, or low-frequency (below detection threshold) heteroplasmic variants pre-existing in the G0 samples that came to predominate by G10. Evidence for a complex mixture of different deletion-bearing mtDNA molecule types present in *C. briggsae* natural strains was provided by a previous analysis of mtDNA in long-term MA lines [19]. A complex mixture of mtDNA deletion products was also observed in a previous analysis of human keratinocytes [20]. Although our findings do not rule out a contribution of high *de novo* mtDNA deletion mutation rates, a transmission bias favoring smaller mtDNA molecules appears to be the main driver of increased *nad5*Δ mtDNA levels at G10 in our study.

The second selfish DNA criterion is that the element must be either neutral or deleterious to the organism encoding it. Previous work has provided strong evidence that *nad5*Δ mtDNA has a negative impact on *C. briggsae* fitness. At the organismal level, heteroplasmic *nad5*Δ levels are negatively correlated with nematode fecundity and pharyngeal pumping rates [10], [12]. At the cellular level, mitochondria from *C. briggsae* strains harboring high levels of *nad5*Δ molecules produce significantly greater ROS levels than those of lower deletion levels [12]. The cumulative body of evidence from these studies indicates that *nad5*Δ molecules have deleterious effects at both the organismal and cellular levels. We thus conclude that *nad5*Δ mtDNA molecules constitute selfish genetic elements.

Despite numerous examples of diverse types of selfish DNA in metazoan nuclear genomes, including the *zeel-1*/*peel-1* pair of linked selfish genes in *C. elegans* [21], [22], examples of selfish mtDNA in animals are more rare. It has been suggested that selfish mtDNA in animals might occur less frequently in animals as compared to plants and fungi due to the ∼10-fold smaller genome size of metazoan mtDNA [4]. Homing endonuclease genes found in the mitochondrial genomes of numerous cnidarian species provide one example of selfish metazoan mtDNA [23]. A transmission bias for smaller mtDNA molecules (containing fewer copies of a noncoding repetitive element) was observed in previous studies of *Gryllus firmus* field crickets [24], [25]. Transmission patterns of the variably sized cricket mtDNA molecules across one generation suggest an average 3% (ranging from −2% to 9%) transmission advantage for smaller molecules relative to larger ones [24]. In this study, we calculated an average 1% (ranging from 0.4 to 1.4% among five strains) transmission advantage for *nad5*Δ mtDNA in *C. briggsae*. In *G. firmus*, the fitness effects of the selfish mtDNA on the organism are unknown, but might be expected to be neutral or very subtle given that no coding sequences are disrupted. In the *C. briggsae* system examined here, however, the *nad5*Δ mitochondrial genomes bear function-disrupting deletions in the *nad5* gene and are associated with deleterious organismal phenotypes [12]. Thus, the differences in average and maximum transmission advantages observed between *G. firmus* and *C. briggsae* might relate to differences in the fitness effects of the selfish mtDNA on ‘host’ organismal fitness.

Why has a selfish genetic element such as *nad5*Δ mtDNA evolved in *C. briggsae*? One hypothesis is that this deleterious mtDNA persists in *C. briggsae* because selection is simply too weak (i.e., population sizes are too small) to eliminate these elements in natural populations. A previous experimental evolution analysis of large deletion-bearing mtDNA in *S. cerevisiae* [3] revealed that high levels of deletion-bearing molecules evolved under small yeast population sizes, but not when yeast population sizes were large and purifying selection was sufficiently strong to prevent deletion accumulation. Further, it is theorized that weak selection associated with long-term evolution in small effective population sizes (*N_e_*) was responsible for the current prevalence of other selfish elements such as transposable elements and introns in the nuclear genomes of multicellular eukaryotes [26]. Thus, it might be expected that *C. briggsae* populations evolving under a larger *N_e_* will be more likely to purge *nad5*Δ mtDNA or evolve compensatory mechanisms as compared to those evolving under a smaller *N_e_*. There is evidence of selection acting against deletions in *C. briggsae* in the form of putative compensatory substitutions in the Ψnad5-2 noncoding element that result in imperfect repeats flanking the deletion region (Figure 1b) and is associated with lower *nad5*Δ mtDNA levels [10]. Two distinct alleles containing such Ψnad5-2 substitutions have been detected in *C. briggsae* natural strains, however both were found in the temperate Clade II (one allele predominantly in North American strains, another allele in predominantly European strains) where there is very little genetic diversity and patterns of silent-site nucleotide diversity suggest a very small *N_e_* of ∼1,000 [10], [13]. Although the *C. briggsae* tropical Clade I harbors much more genetic diversity (*N_e_* estimated to be ∼63,000), no Ψnad5-2 compensatory substitutions have been detected in dozens of geographically diverse strains from this clade [11]. It remains unclear why the observed evolutionary patterns conflict with theoretical expectations. It is possible that the *nad5*Δ mtDNA is sufficiently more deleterious in the temperate Clade II strains and/or environments such that selection acts on it. It is also possible that tropical Clade I strains have evolved different compensatory mechanisms (e.g., through cyto-nuclear epistasis) that are more difficult to identify.

Another hypothesis for the evolution of selfish mtDNA in *C. briggsae* is that the androdioecious reproductive mode (self-fertile hermaphrodites and males) of this species provides an evolutionary environment that favors selfish mtDNA proliferation. In *C. briggsae* (and its more famous congener *C. elegans*), two reproductive strategies are possible. Hermaphrodites (XX) are capable of self-fertilization to produce progeny that are almost always hermaphrodites (except in those rare instances where males are spontaneously produced through X chromosome nondisjunction). Males (X0), when present, are capable of fertilizing hermaphrodites thus allowing their sperm to compete with sperm of hermaphrodite origin for egg fertilization. Like most other animals, mtDNA is usually observed to be inherited through the maternal (hermaphrodite) lineage in *C. briggsae*, though there is indirect evidence for paternal leakage of mtDNA in certain experimental crosses (D. R. D., unpublished data). Thus, in hermaphroditic reproduction virtually 100% of the progeny will bear mtDNA capable of being transmitted to the next generation. Outcrossing, by contrast, results in progeny where a much smaller fraction (∼50% if there is no sex ratio distortion) of the progeny bear mtDNA capable of subsequent transmission. *C. briggsae* hermaphroditic reproduction is therefore much more favorable than outcrossing, from a selfish mtDNA perspective.

We also note that the variation among MA line-specific G0 progenitors (each initiated from the self-progeny of a common parental hermaphrodite for each strain analyzed) in terms of *nad5*Δ mtDNA levels (Figure 3) reveals that there was variation among MA line progenitors in terms of starting *nad5*Δ levels. This observation is significant in that it undermines a common assumption of MA experiments that all replicate lines start off being genetically identical. In most nematode MA experiments, focal strains are generally bottlenecked in the lab for 5−10 generations prior to the establishment of MA lines for the purpose of eliminating nuclear heterozygosity prior to the experiment [27], [28]. Given the results of this study, it would be expected that this “pre-MA bottlenecking” treatment will lead to the accumulation of higher *nad5*Δ mtDNA levels at the onset of MA experiments. A previous analysis of a separate long-term set of *C. briggsae* HK104 and PB800 MA lines revealed that the HK104 MA line progenitor strain had evolved higher *nad5*Δ mtDNA levels as a consequence of pre-MA bottlenecking whereas the PB800 progenitor strain deletion levels were similar to pre-bottlenecking levels [19]. The results presented here support the previously observed pattern, as the HK104 MA lines analyzed here experienced higher incidences of *nad5*Δ mtDNA accumulation as compared to the PB800 lines (Figure 3). It is possible that the accumulation of high *nad5*Δ mtDNA levels in pre-MA bottlenecked *C. briggsae* MA lines might contribute to the observed faster rate of fitness decay in this species as compared to *C. elegans* [27]. However, *C. briggsae* also experiences higher mutation rates at nuclear microsatellite loci [29] and in terms of *de novo* heteroplasmic mtDNA deletion formation [10] as compared to *C. elegans*, so the high *nad5*Δ mtDNA levels in MA line progenitors is not the sole underlying source of fitness decay in *C. briggsae* MA lines.

Our characterization of *C. briggsae nad5*Δ mtDNA provides a novel and powerful system for studying selfish mtDNA in animals. Although the transmission bias of *nad5*Δ mtDNA molecules demonstrated in this study, coupled with previous characterizations of deleterious phenotypes associated with high deletion levels provides sufficient evidence that these elements constitute selfish DNA, more work is required to elucidate the evolutionary dynamics of this system. The extent to which selfish mtDNA threatens other animal species is also unknown. We speculate that selfish mtDNA in animals, though sometimes characterized as rare [4], might be more widespread (particularly in species where self-reproduction occurs), but has remained a cryptic phenomenon owing to difficulties in discovering heteroplasmic selfish mtDNA variants, demonstrating transmission advantages, and characterizing phenotypic effects of particular mtDNA elements.

## Materials and Methods

### Propagation and Maintenance of Experimental Lines

MA lines were propagated from the eight *C. briggsae* natural strain progenitor strains indicated in Fig. 1a. For each of the eight progenitor strains, 24 replicate MA lines were initiated as described in the Results and passaged through single-nematode bottlenecks as previously described [27]. MA lines were propagated on NGM agar plates with *Escherichia coli* OP50 as a food source at 20°C, as in previous nematode MA experiments. Unlike most previous nematode MA experiments, we did not inbreed the progenitor strains (usually eight generations of inbreeding) prior to establishment of MA lines. We previously gained evidence that the *nad5*Δ mtDNA levels might be highly plastic based on an analysis of mtDNA in a separate set of long-term (250-generation) MA lines derived from *C. briggsae* HK104 and PB800, and did not want to inflate deletion levels by bottlenecking (usually for eliminating heterozygosity at nuclear loci) prior to MA experiments. For each of the eight strains included in the analysis, one single hermaphrodite nematode was allowed to self-reproduce, after which 24 individual F_1_ hermaphrodite progeny were individually picked to new plates to serve as the G0 nematodes used to initiate a specific MA line.

After the G0 nematodes self-reproduced and single nematodes were transferred to new plates to create the new G1 generation, the remaining F_1_ progeny on the G0 plate were allowed to undergo one round of reproduction (creating F_2_ progeny), yielding many thousands of nematodes which were harvested and used for DNA extractions (performed using standard proteinase K-based lysis buffer methods) to be used in our PCR assays. The same procedure was used for G10 nematodes – the nematodes selected to initiate G10 were allowed to self-reproduce for two generations, after which nematodes were harvested for DNA extraction and subsequent PCR. Fractions of the nematodes collected to represent G0 and G10 were also cryogenically preserved using standard protocols [30].

### PCR, DNA Sequencing, and Statistical Analysis

We performed the standard PCR analysis of the *nad5* deletion locus using the Cb_mt1F and 58R primers and thermal cycling parameters previously described [10]. PCR products were electrophoresed on standard agarose gels and digitally recorded as *.TIF files using a UV-TMP gel documentation system. Gel images were manually scored according to the three categories discussed in the Results (intact = large band only; intermediate  = both bands; deletion = small band only). The correlation between our standard PCR approach and the qPCR approach used in Denver and Howe 2008 is discussed in the Results.

For DNA sequencing analysis, we selected 17 individual MA lines that were scored as having the intermediate phenotype at G0 and the deletion phenotype at G10. We repeated the PCR band assay and purified the resulting bands. For the G0 samples, we excised the lower deletion band from the gel and purified the small amplicon DNA with a QIAquick gel extraction kit. For the G10 samples, we used a magnetic bead cleanup approach. While the PCR reactions were successful for the G0 DNA samples, we were unable to obtain enough purified product for sequencing for most reactions. We sequenced the deletion products using the 58R primer used for the original PCR amplification using BigDye with standard reaction conditions (Applied Biosystems). Sequences were aligned to the AF16 reference sequence in Genbank (AC186293.1) to characterize deletion boundaries.

We carried out statistical assessment of our categorical band scoring data using a two-way ANOVA approach in R. Because the number of MA lines analyzed per strain varied between 23 and 24 (Table 1), we reduced the number of lines included in the ANOVA to 23 for all six Clade I and II *C. briggsae* strains analyzed to balance the data set. The line selected for removal from analysis (for those two strains where necessary) was determined using an online random number generator.
